# Changes in femoral rollback and rotation with increasing coupling in knee arthroplasty—a biomechanical in-vitro study

**DOI:** 10.1186/s12891-023-06430-w

**Published:** 2023-05-02

**Authors:** Andrea Lorenz, Alexander Winter, Moritz Mederake, Clemens Freidhager, Ulf Krister Hofmann, Ulf Gunther Leichtle

**Affiliations:** 1 Austrian Center for Medical Innovation and Technology (ACMIT Gmbh), Wr. Neustadt, Austria; 2 Department of Orthopaedic Surgery, University Hospital Tübingen, Hoppe-Seyler-Straße 3, 72076 Tübingen, Germany; 3 Institute for Lightweight Design and Computational Biomechanics, TU Wien, Vienna, Austria; 4 Department of Trauma and Reconstructive Surgery, BG Klinik, University of Tübingen, 72076 Tübingen, Germany; 5 Department of Orthopedic Trauma and Reconstructive Surgery, University of Aachen Medical Center, Pauwelsstraße 30, 52074 Aachen, Germany; 6 Practice for Orthopaedic, Spine and Trauma Surgery, Rottenburg, Germany

**Keywords:** Femoral rollback, Total knee arthroplasty, Hinged knee, Knee simulator, Biomechanics

## Abstract

**Background:**

After total knee arthroplasty, 10–30% of patients still complain about knee pain, even after exact positioning of the components. Altered knee kinematics are crucial in this regard.

The aim of our study was to experimentally determine the influence of different degrees of component coupling of knee prostheses on joint kinematics during muscle-loaded knee flexion in-vitro.

**Methods:**

Femoral rollback and femoral rotation of a standard cruciate retaining (GCR), a posterior stabilized (GPS), a rotational hinge (RSL) and a total hinge (SSL) design of the same series of knee replacement implants (SL-series) of one single manufacturer (Waldemar Link GmbH, Hamburg, Germany) were analyzed and set in relation to the motion of the corresponding native knee in a paired study design. All different coupling degrees were analyzed in the same human knees. To simulate muscle loaded knee flexion, a knee simulator was used. Kinematics were measured with an ultrasonic motion capture system and integrated in a calculated coordinate system via CT-imaging.

**Results:**

The largest posterior motion on the lateral side was found for the native knee (8.7 ± 7.0 mm), followed by the GPS (3.2 ± 5.1 mm) and GCR (2.8 ± 7.3 mm) implants, while no motion was found for the RSL (0.1 ± 3.0 mm) and the SSL (-0.6 ± 2.7 mm) implants. In contrast, on the medial side, only the native knee showed a posterior motion (2.1 ± 3.2 mm). Regarding femoral external rotation, the only implant where the observed difference did not reach statistical significance when compared to the native knee was the GCR (*p* = 0.007).

**Conclusion:**

The GCR and GPS kinematics closely imitate those of the native joint. Medial femoral rollback is reduced, however, with the joint pivoting around a rotational center located in the medial plateau. Without additional rotational forces, the coupled RSL and SSL prostheses closely resemble each other with no femoral rollback or relevant rotational component. The femoral axis, however, shifts ventrally in both models when compared with their primary counterparts. The positioning of the coupling mechanism in the femoral and tibial component thus can already lead to altered joint kinematics even in prostheses with an identical surface geometry.

## Background

Primary total knee arthroplasty (TKA) is a successful procedure which is performed over 170,000 times each year in Germany alone with still increasing numbers [1]. Despite improving devices and surgical techniques, about 10–30% of patients still complain about knee pain even after exact positioning of the components [2, 3]. While the reasons for these unsatisfactory results are not yet fully understood, the persisting pain is largely attributed to altered kinematics of the implanted device in comparison to a healthy knee joint [4]. Other reasons for persisting pain can be aseptic loosening of the implant, periprosthetic joint infection, or fracture [1]. In these latter cases, revision surgery is necessary which in many cases entails an increase in the grade of coupling of the newly implanted device.

Depending on the surgical needs, there are different implant types available with different degrees of coupling. These also influence joint kinematics to different degrees. The standard uncoupled, posterior-cruciate ligament retaining joint surface replacement allows motion in all 6 degrees of freedom. Nevertheless, joint kinematics are still altered due to the modified joint surfaces as well as the resection of the anterior cruciate ligament [5]. If the posterior cruciate ligament is damaged as well, an uncoupled but posterior stabilized primary knee endoprosthesis can be implanted, which additionally restricts posterior motion of the femur with respect to the tibia [6]. In contrast, rotational hinge or total hinge prostheses, which are mostly used in revision surgery, are coupled devices that restrict motion to 2 degrees or even just 1 degree of freedom. Common indications are instability, bone loss, aseptic loosening and infection [7–9]. These implants take over the function of all ligaments which can thus be resected, if not already damaged preoperatively. Knee kinematics are then primarily determined by the implant itself, only allowing a uniradial flexion/extension around a fixed, predefined axis with the total hinge. In a rotational hinge prothesis, the one-dimensional flexion/extension movement is supplemented by the possibility for internal/external rotation of the tibia, especially in flexion. The increase in the degree of coupling is generally considered to be associated with an increase in aseptic loosening of the implant. The idea behind that concept is, that in hinged knees forces acting on the knee can not be attenuated by the deformation of soft tissues any more but they directly act on the bone-implant interface. For this reason, knees with a rotational or a total hinge are routinely implanted with additional stem fixation, which have been shown to be beneficial for implant survival [10–12]. With respect to functional outcome, the available data are controversial and in large parts of a retrospective nature. Some authors warn about a high complication rate and low survivorship of such coupled implants [13], while others report encouraging outcomes when using newer implant designs [14, 15]. Also the underlying biomechanics and kinematics still remain poorly understood.

Over the last decades, technical advances like mobile fluoroscopy and implants equipped with sensors have much facilitated the analysis of in-vivo knee kinematics without and with different types of knee prostheses [16–18]. While these in-vivo methods can measure knee kinematics for realistic loading situations (e.g. gait, deep knee bend, stair climbing etc.) with improved accuracy compared to traditional methods like gait analyses based on optoelectronic measurements [19], they can only provide a statistical comparison of different implant types averaged across different patients. The influence of multiple factors of implant design can also partly be analyzed in-silico, using multi-body and finite-element models [20, 21]. While these techniques allow to analyze the impact of patient-, surgical- and implant design-specific factors separately, they rely on mathematical models with many estimated parameters, which mostly are only validated for a few specific cases using in-vitro measurements. In contrast, in-vitro biomechanical studies, using e.g., an Oxford-rig-like knee simulator, can provide the possibility to directly test multiple implants on the same knee with realistic muscle and soft-tissue tensions. Beside some system dependent weaknesses (e.g. reduced muscle force, quasi-static conditions), they thus allow to directly compare their effects on knee kinematics in a system resembling closely the physiological condition [22]. Arnout et al. for example compared eight different posterior-stabilized TKA designs in an in-vitro study on a knee testing rig. To be able to compare different types of prostheses in the identical knee specimen without changes in kinematics after prior implantations, they did not use real knees but an artificial mechanical model of the knee joint [22]. Another key issue is that of varying joint surface geometries of different providers. When analyzing the effect of different degrees of coupling on knee kinematics, only implants of the same provider with identical joint surface geometries may be used to not confound the changes caused by varying geometries with the effects of different degrees of coupling.

The aim of our study was to experimentally determine the influence of different degrees of component coupling of knee prostheses on joint kinematics during muscle-loaded knee flexion in-vitro. Femoral rollback and femoral rotation of a standard cruciate retaining, a posterior stabilized, a rotational hinge and a total hinge design of the same series of knee replacement implants of one single manufacturer (Gemini and Endo-Modell SL-series, Link, Hamburg, Germany) were analyzed and set in relation to the motion of the corresponding native knee in a paired study design. All different degrees of coupling were analyzed in the same human knee.

## Methods

Tibiofemoral joint kinematics of different degrees of coupling were measured in 10 human cadaveric knees during an in-vitro simulation of a muscle loaded knee flexion using an established knee simulator [23–26]. The study was conducted according to the guidelines of the Declaration of Helsinki, and was approved by the local ethics board of the University Hospital of Tübingen (registration number 304/2016BO2). Permission for using the specimens was given by the local ethics board of the University Hospital of Tübingen (Ethik-Kommission – Universitätsklinikum Tübingen). Informed consent and consent to participate was not applicable in case of cadaveric specimens. The cadaveric specimens were acquired from Science Care (Phoenix, AZ, USA), where informed consent was given prior to death. Science care is accredited by American Association of Tissue Banks.

### Knee specimens and preparation technique

Ten fresh-frozen left human cadaveric knees were used (Science Care, Phoenix, AZ, USA). There were seven female and three male specimens with an age of average age of 71.3 (SD 15.7) years. Prior to the measurements, the specimens were thawed for 12 h at room temperature. The skin and subcutaneous soft tissue were removed, leaving the joint capsule around the knee joint intact plus 5 major muscle tendons (vastus lateralis, rectus femoris, vastus medialis, biceps femoris, and semimembranosus). The tibia and femur were cut 15 cm from the joint line and the fibula was screwed to the tibia. To compensate for the influence of the capsular suture on the results when comparing the native knee with the different types of coupling, the joint capsule was already opened and sutured again prior to the native measurement. At this occasion, the joint was also examined. Specimens with damaged ligaments (including the anterior cruciate ligament), severe osteoarthritis or prior knee surgery were excluded. Computed tomography (CT) scans (Somatom Definition AS, Siemens Healthcare GmbH, Erlangen, Germany) and X-rays (GE Lunar DPX-L, GE Healthcare, Chicago, IL, USA) were additionally performed. During the measurements, the specimens were sprayed with saline solution at regular intervals and loosely wrapped into thin plastic foil to keep them moist.

### Knee simulator

**Fig. 1:**
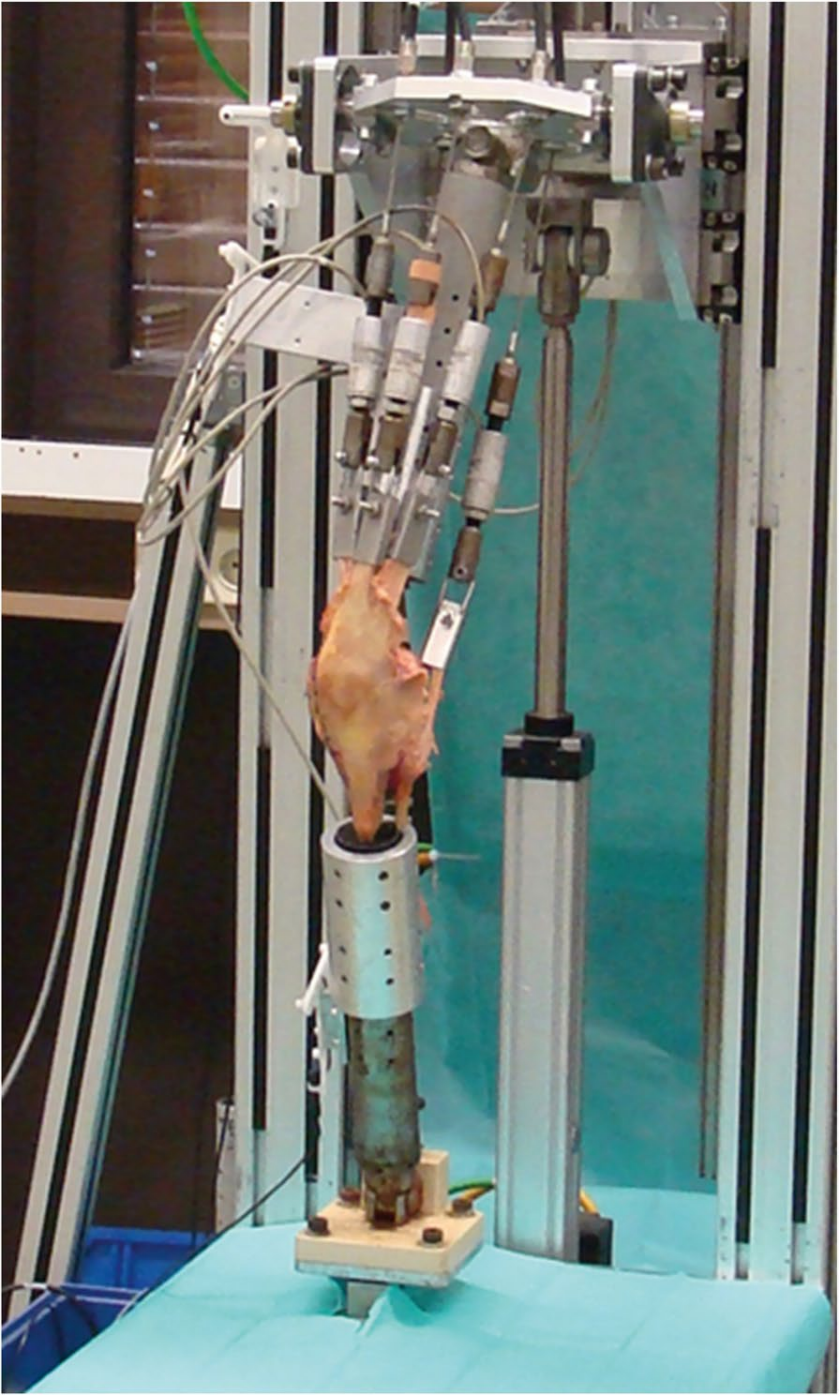
Knee simulator. Quasistatic, muscle-loaded knee flexion was simulated on 10 human knee specimens. The tendons of vastus medialis, vastus lateralis, rectus femoris and semimembranosus were clamped to the knee simulator and individually controlled

To simulate muscle loaded knee flexion, a knee simulator [23–26] was used (Fig. 1). The tibia and femur were fixed at the osteotomy sites to the ankle and hip assembly using aluminium cylinders and the five muscle tendons were connected to servo motors via custom-made tendon clamps. Muscle loaded knee flexion was simulated from 15° to 90° of flexion. As known from preliminary tests, the movement cycle could not be started with extension, since the electromechanics could not control the direction of movement at 0° flexion. To avoid damage to the specimens due to hyperextension, the movement cycles therefore always began at 15° flexion. During the downwards motion of the hip assembly at a flexion velocity of approximately 0.5°/sec, the quadriceps forces were controlled to achieve a constant vertical ankle force of 50 N. To account for physiological muscle tension of the antagonists, the hamstring forces were kept constant at 20 N. This is equivalent to a quasi-static squatting movement, where the vertical ankle force equals the body weight. To prevent tendon rupture, only a reduced force level was simulated. An equal load distribution between the 3 parts of the quadriceps muscle was used. Two flexion – extension cycles per implant were performed for kinematic measurement. Details on the knee simulator are given in Müller et al. (2009) [26].

### Kinematics measurements

During the simulated knee flexion, kinematics of the femur, tibia and patella were measured with an ultrasonic motion capture system (ZEBRIS CMS-HS, Isny, Germany) at a sample rate of 1 Hz. Two marker triads fixed at the tibia and femur as well as a static transmitter unit were used for data collection (resolution: 0.085 mm, accuracy: 1 mm). To define reproducible coordinate systems (CSs), two reference points were marked on each bone using screws: the most prominent points of the medial and lateral femoral epicondyles, and medially and laterally 5 cm distal to the most prominent points of the tibial plateau. These points were recorded prior to the measurements. The initial CSs were defined according to Lorenz et al. [25], which is based on Grood and Suntay [27].

### Knee conditions and implantation technique

**Fig. 2:**
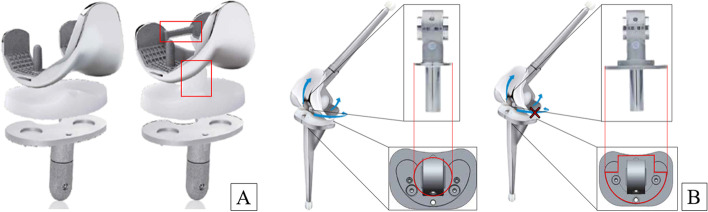
Different degrees of coupling of the Gemini and Endo-Modell SL series (Waldemar Link GmbH, Hamburg, Germany) (with the kind permission of Waldemar Link GmbH). **A** Uncoupled prostheses without (left image) and with (right image) posterior stabilized modification (marked in red). **B** Coupled prostheses with rotational hinge (left) and total hinge (right) and their coupling mechanisms. In the rotational hinge model, the collar (marked in red) is rotationally mobile whereas in the total hinge model the collar is fixed with two screws to impede rotational movement

Measurements were performed on the native knee and the knee after implantation of a cruciate retaining (GCR), a posterior stabilized (GPS), a rotational hinge (RSL) and a total hinge (SSL) prosthesis of the same series (Gemini and Endo-Modell SL series, Waldemar Link GmbH, Hamburg, Germany) (Fig. 2). To analyze just the effect of the different coupling degrees, a fixed bearing inlay was chosen for the GCR and GPS knee.

The surgical procedure for all experiments was performed by the same experienced surgeon, according to the company's surgical instructions. A modified anteromedial approach was used, leaving the prepared quadriceps tendons intact to prevent tendon rupture during the experiments. Bone preparation was done using an intramedullary positioning of the femoral cutting guide, as well as a combined intra- and extramedullary positioning of the tibial cutting guide. The size was determined using the original femur size gauge. The gauge was aligned with the dorsal condyles, three degrees of femoral external rotation was set, and care was taken to ensure that the ventral cut was level with the anterior femoral cortex to avoid overstaffing and notching. The implants were cemented carefully, in order to guarantee stability during the measurements, but to also allow easy revision. The inlay size was not constant between the individual patients, but was adapted to the individual ligament tension to ensure stable collateral ligament guidance. The chosen inlay size for a specimen was, however, constant among the GCR and GPS implant. Prior to revision surgeries, the exact positioning of the femoral and tibial implants was marked. Due to the use of an associated series of implants, revision surgeries could be carried out easily in only a few steps. The collateral ligaments were released prior the implantation of hinged implants. Care was taken to position the implants at exactly the same position as the initial primary GCR knee.

### Determination of the cylindrical axis and calculation of femoral rollback and rotation

**Fig. 3:**
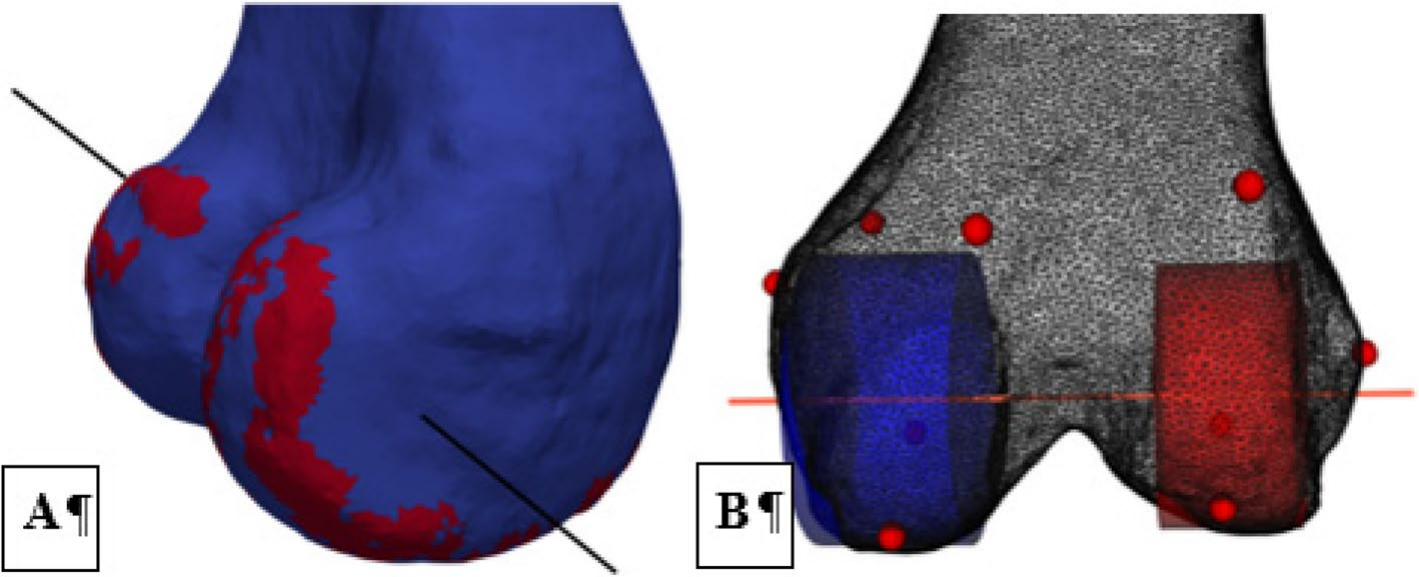
Determination of the femoral axis. **A** A cylindrical axis was defined by identifying the femoral condyles as geometrical ridge-regions (red) using a curvature-based algorithm (Subburaj, Ravi [33] and (**B**) subsequent fitting of spheres [22] (red: medial sphere; blue: lateral sphere)

In in-vivo fluoroscopy studies, anterior–posterior motion (femoral rollback) and the distribution of this rollback on the medial and lateral compartment of the knee (femoral rotation) are mostly measured by tracking the most distal points of the medial and lateral femoral condyles (potential contact point) and projecting them onto the tibial plateau [16, 18]. In contrast, the most common description used in biomechanical in-vitro studies, like in the current study, is based on the technique proposed by Grood and Suntay [27] defining a femur-fixed flexion–extension axis (e.g. the transepicondylar axis), a tibia-fixed internal–external rotation axis (e.g. the tibial shaft axis) and a floating anterior–posterior axis resulting from an Euler-angle-like mathematical description. While this is mathematically correct and allows a good comparison of in-vitro knee kinematics, obtained data are difficult to interpret from a clinical point of view. For this purpose, Victor et al. [28] proposed an alternative method using the cylindrical femur axis described by Eckhoff et al. [29] as the flexion–extension axis and projecting it onto the tibial plateau to describe medial and lateral femoral rollback. In an magnet resonance imaging (MRI) study, Pinskerova et al. [30] could demonstrate that this projection of the cylindrical femur axis is in good accordance with the contact point and, as such, the line connecting the most distal points of the medial and lateral femoral condyles used in in-vivo studies or most of the flexion range. To determine the cylindrical femur axis, however, requires imaging of the knees time-consuming post-processing. For this purpose, we developed a new method to image-based calculate the cylindrical femur axis, the femoral rollback on the medial and lateral side, as well as femoral rotation. Taking the findings of Pinskerova et al. [30] into account, this allows us to compare our results with those of in-vivo fluoroscopy studies. To determine the cylindrical axis, the CT-scans of the native knee joints were segmented using Medtool (Dr. Pahr Ingenieurs, Pfaffstätten, Austria) and 3D Slicer [31, 32] and stl-files of the bone surfaces were provided semi-automatically. An algorithm for the curvature-based landmark detection [33] was adapted and applied to the stl-data of the femoral condyles identifying the edges of the condyles as geometric ridge-regions based on the geometric surface curvature [34]. Subsequently, spherical fits were computed to each condyle. The centers of the spheres were defined as condyle centers and the connecting line as cylindrical femur axis according to the definition of Eckhoff et al. [29] (Fig. 3).

To calculate the femoral rollback, new CSs were defined: For the femur, the flexion–extension axis of the initial CS was replaced by the calculated cylindrical axis. At the tibia, the most lateral and medial points of the native tibial plateau were identified in the CT-scans and replaced the mediolateral axis at the tibial CS. The screws used as a reference for the kinematics measurements were determined in the CT-scan as well. This allowed subsequent coordinate transformations from the initial bone CS to the newly defined CS based on the CT data. The medial and lateral condyle centers were projected onto the tibial plateau and their anterio-posterior i.e. sagittal motion during the measured knee flexion was defined as medial and lateral femoral rollback. In addition, the rotation of the cylindrical femur axis with respect to the mediolateral axis of the tibial plateau was calculated as femoral axial rotation.

### Statistical analysis

All measured data were resampled in one-degree steps and illustrated with respect to the tibiofemoral flexion angle. Normality of the data was assessed by histograms and a parametric approach was chosen. Repeated measures analysis of variance (ANOVA) was performed to compare the differences between the different implant types at the 95% confidence level (α = 0.05). Post-hoc tests were performed by means of paired t-test using a Bonferroni corrected α_corr_ = 0.005 for multiple testing, resulting in ten comparisons. Statistical significance was computed for the 20° position (starting position where all conditions on all knees could be measured) and for the 85° (final position where all conditions on all knees could be measured). Graphic illustrations were performed by line diagrams, the error bars indicating the standard deviation. Projections of the cylindrical axis between the medial and lateral condyle on the tibial plateau were calculated for different flexion angles to better illustrate the characteristics of the different conditions. All calculations were performed using Python 2.7 [35].

## Results

**Fig. 4:**
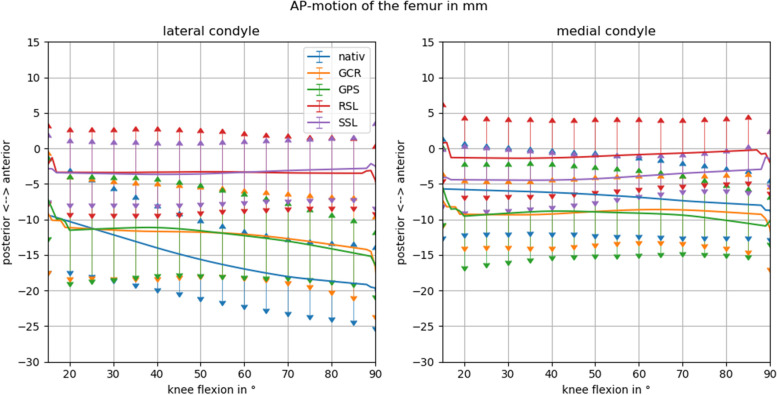
Anteroposterior (AP) motion from extension to flexion of the lateral (**A**) and medial (**B**) femoral condyles with respect to the tibial plateau for the 4 different types of implants compared to the native knee. A strong femoral rollback can be observed in the native knee. This rollback is partially imitated by the cruciate ligament retaining (GCR) and posterior stabilized (GPS) primary knees. No lateral femoral rollback can be observed in the rotating hinge (RSL) and total hinge knee (SSL). On the medial side the native knee also shows a discrete femoral rollback during flexion. This is, however, not imitated by any of the investigated prothesis. Of note, the position of the femoral axis with respect to the tibial axis in the sagittal plane of the hinged knees appears more ventrally throughout all degrees of flexion

Comparing femoral rollback of the different implant types, the largest posterior motion on the lateral side was found for the native knee (8.7 ± 7.0 mm), followed by the GPS (3.2 ± 5.1 mm) and GCR (2.8 ± 7.3 mm) implants, while no motion was found for the RSL (0.1 ± 3.0 mm) and the SSL (-0.6 ± 2.7 mm) implants (Fig. 4, Table 1).

**Table 1:** Femoral rollback and femoral external rotation of the different implant types from 20° of flexion to 85° of flexion

	**Femoral rollback**		**Femoral external rotation mean (SD) [°]**
	**Lateral condyle mean (SD) [mm]**	**Medial condyle mean (SD) [mm]**	
**native**	8.7 (7.0)	2.1(3.2)	8.1 (6.9)
**GCR**	2.8 (7.3)	-0.1 (4.4)	3.4 (7.8)
**GPS**	3.2 (5.1)	1.0 (4.5)	2.5 (5.3)
**RSL**	0.1 (3.0)	-1.0 (3.6)	1.3 (4.2)
**SSL**	-0.6 (2.7)	-1.4 (5.0)	0.8 (3.8)

*Abbreviations*: *SD* standard deviation; native—native knee, *GCR* Gemini cruciate retaining, *GPS* Gemini posterior stabilized, *RSL* rotational hinge model, *SSL* total hinge model

The values of these latter two implant types also differed statistically significantly from the native knee and the GCR/GPS prostheses, while the difference between the two primary designs and the native knee did not reach statistical significance (Table 2).

**Table 2:** Comparison of the femoral rollback at 85° flexion

	**native knee**	**CR**	**PS**	**RSL**
lateral condyle				
CR	0.046			
PS	0.023	0.645		
RSL	** < 0.005**	** < 0.005**	** < 0.005**	
SSL	** < 0.005**	** < 0.005**	** < 0.005**	0.612
medial condyle				
CR	0.514			
PS	0.250	0.318		
RSL	** < 0.005**	** < 0.005**	** < 0.005**	
SSL	0.038	0.011	**0.005**	0.097

Statistically significant p-values on the base of a Bonferroni adjusted alpha (α_corr_ = 0.005) are depicted in bold

*Abbreviations*: *GCR* Gemini cruciate retaining, *GPS* Gemini posterior stabilized, *RSL* rotational hinge model, *SSL* total hinge model

In contrast, on the medial side, only the native knee showed a posterior motion (2.1 ± 3.2 mm) while this medial femoral rollback remained negligible for all four tested implants (< 1.5 mm) (Table 1).

**Fig. 5:**
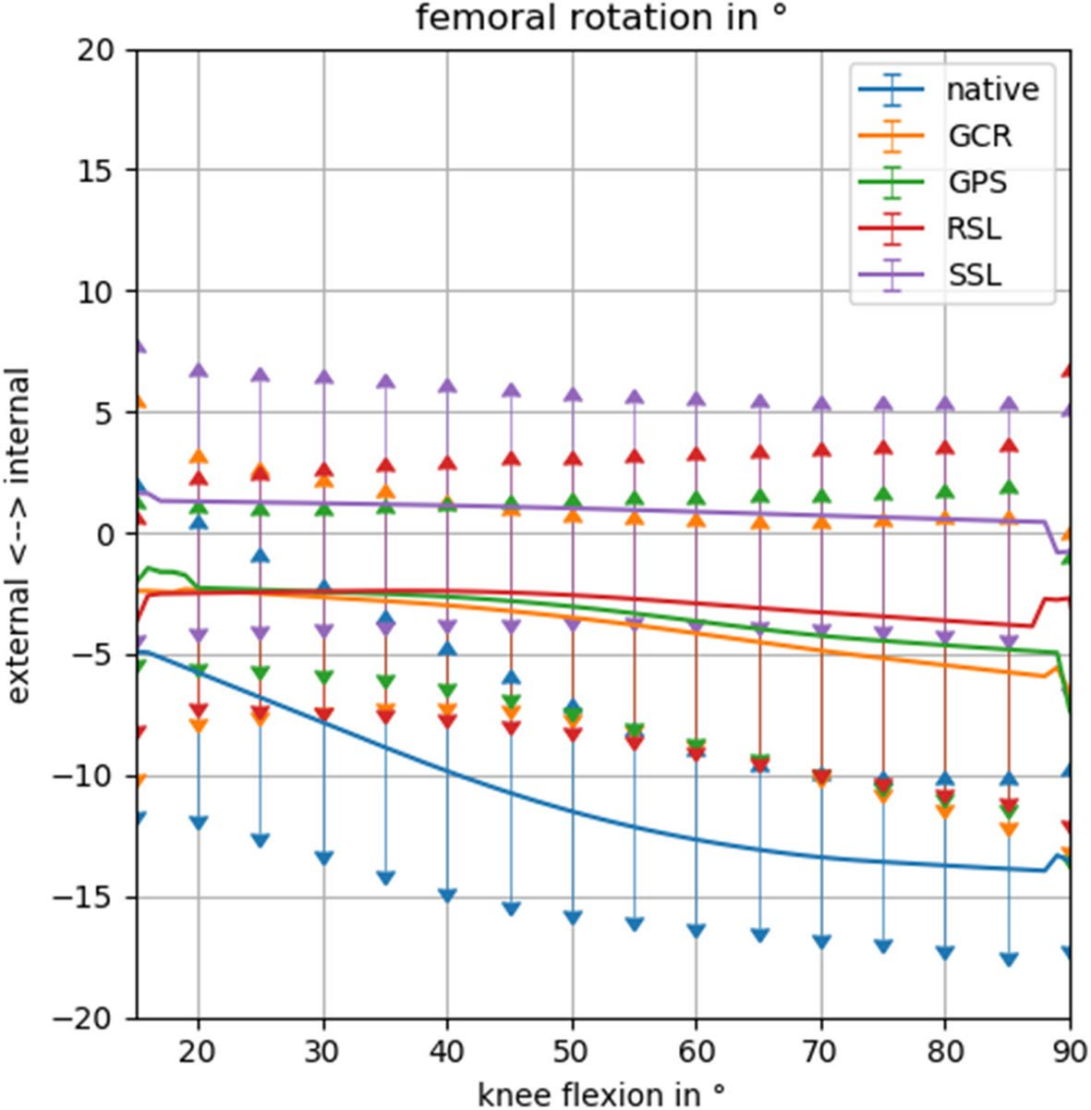
Rotation of the cylindrical femoral axis with respect to the tibial plateau, comparing the 4 different implant types to the native knee. In the native knee, the characteristic external rotation of the femoral axis on the tibia can be observed from extension to flexion. This external rotation can also be noted in the cruciate ligament retaining (GCR) and posterior stabilized (GPS) knee and to an even lesser extent in the rotating hinge knee (RSL). No such external rotation was seen in the total hinge knee (SSL)

Regarding femoral external rotation, the knee with the SSL implant already started in a 1.3° (± 4.9°) internally rotated position, while the other implants (~ 2.5°) and the native knee (5.8° ± 5.6°) were slightly externally rotated. During knee flexion, external rotation was negligible for the SSL (0.8 ± 3.8°) and RSL prostheses 1.3° (± 4.2°). However, even the two primary designs showed a strongly reduced external femoral rotation with 2.5° (± 5.3°) for the GPS and 3.4° (± 7.8°) for the GCR knee in comparison to the native knee with 8.1° (± 6.9°) (Fig. 5, Table 1). The only implant where the observed difference did not reach statistical significance when compared to the native knee was the GCR (*p* = 0.007) (Table 3).

**Table 3:** Comparison of external femoral rotation at 85° flexion

	**native**	**CR**	**PS**	**RSL**
CR	0.007			
PS	** < 0.005**	0.496		
RSL	** < 0.005**	0.127	0.399	
SSL	** < 0.005**	0.028	0.015	0.118

Statistically significant p-values on the base of a Bonferroni adjusted alpha (α_corr_ = 0.005) are depicted in bold

*Abbreviations*: *GCR* Gemini cruciate retaining, *GPS* Gemini posterior stabilized, *RSL* rotational hinge model, *SSL* total hinge model

**Fig. 6:**
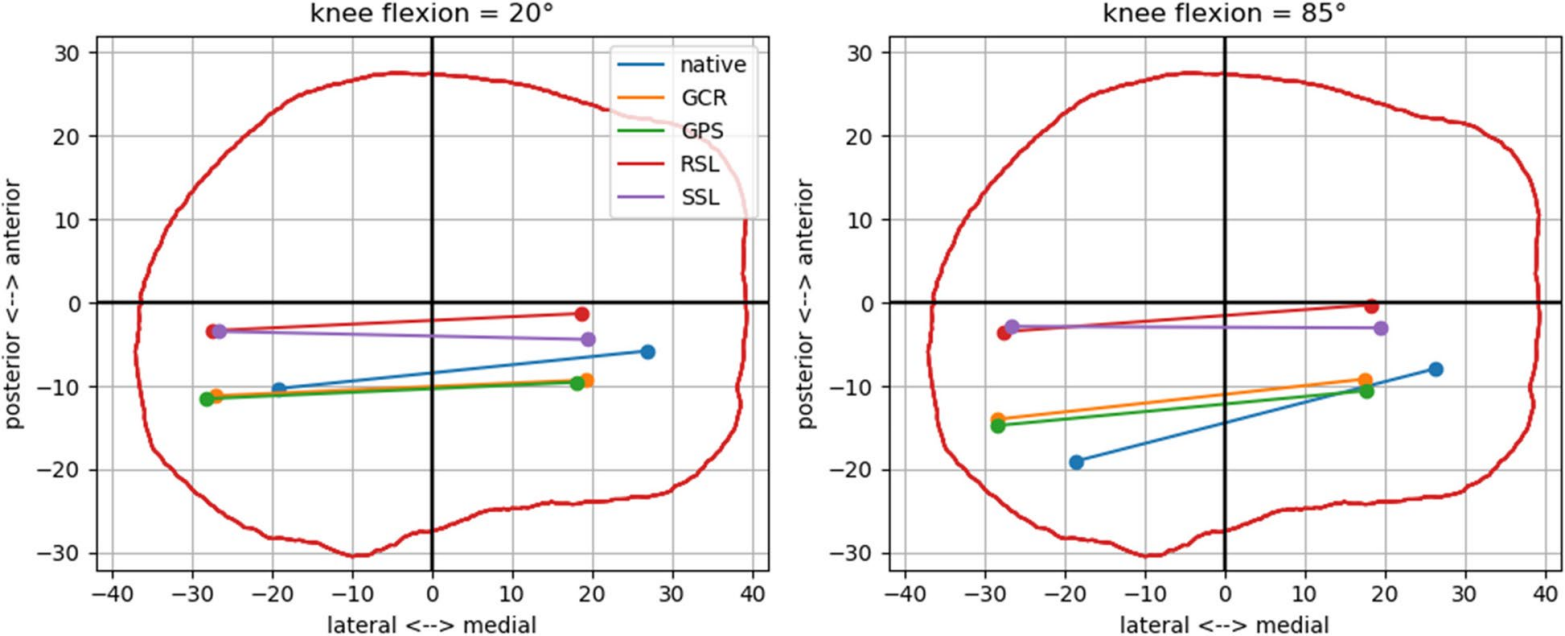
Projections of the cylindrical femur axes onto the tibial plateau for the different implants in 20° (**A**) and 85° (**B**) of flexion. In 85° of flexion, the externally rotated and posteriorized position of the femoral axis can be seen in the native knee. The axis position of the cruciate ligament retaining (GCR) and posterior stabilized (GPS) primary knees approximate this position without, however, being able to fully imitate the external rotation and femoral rollback of the native knee. The axes of the rotating hinge knee (RSL) and the total hinge knee (SSL) are located more ventrally in both positions without showing any relevant movement from extension to flexion

**Fig. 7:**
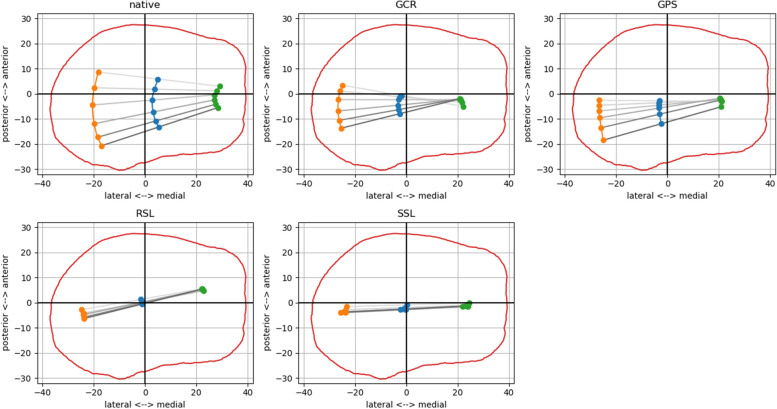
Projections of the cylindrical femur axis onto the tibial plateau for different flexion angles (extension: light grey, flexion: dark grey) for the different implants of a single knee specimen. In the native knee the characteristic posterior femoral rollback can be observed with increasing knee flexion. This rollback is much more pronounced laterally than medially. This movement is partially imitated by the cruciate ligament retaining (GCR) and posterior stabilized (GPS) primary knees. In both systems, however, a real rollback is not observed medially, but rather the lateral rollback pivots around a medial turning point. In the rotating hinge knee (RSL) and the total hinge knee (SSL) no femoral rollback can be measured, but the axis position remains constant through all flexion angles. Of note, the sagittal axis position is located more ventrally than in the primary conditions

The starting and end positions with the different implants can be better visualized by projecting the cylindrical femur axis onto the tibial plateau. The axis positions of GCR and GPS were almost identical to the native knee. However, in the coupled prostheses, the projected axes lay more ventrally than in the native knee, the GCR and GPS. This difference was slightly more prominent for higher flexion angles, since the coupled implants did not allow femoral rollback (Figs. 6 and 7).

## Discussion

In the present study, tibiofemoral joint kinematics of different coupling grades of the same implant series were measured on human cadaveric knees in a controlled laboratory in-vitro study using a well-established knee simulator [23–25, 36]. A standard cruciate retaining, a posterior stabilized, a rotational hinge and a total hinge prosthesis were compared to the native knee regarding medial and lateral femoral rollback as well as external femoral rotation.

Our presented study design allowed to perform several revision operations and testing with increasing coupling on the same knees, thus leading to a highly stringent data set.

When comparing joint kinematics of the uncoupled GCR and the GPS almost no difference could be observed regarding femoral rollback and rotation. Both models principally imitate the joint kinematics of the native knee with an external femoral rotation during flexion, mostly generated by a relevant lateral femoral rollback. In absolute terms, these movements are, however, slightly reduced in comparison to the native knee. The most notable difference is the lack of a medial femoral rollback after TKA so that the lateral knee effectively pivots around a rotational center located in the center of the medial tibial plateau. Since only the anterior cruciate ligament is resected for the GCR implant, such a difference is quite interesting. Of note, in anterior cruciate ligament-deficient knees, a similar observation was made: the center of rotation appears to shift to the medial compartment [37]. If this is the only reason why the center of rotation after implantation shifts, remains unclear. Another possible explanation for this observation is the high conformity that is typical for the GCR and GPS inlay design in contrast to the relatively flat native tibia [38]. Such a high conformity might, however, also be necessary to compensate for the lack of the anterior cruciate ligament, especially if the physiological slope of the tibia is imitated during implantation of the device. To date, it remains unknown which design provides the best joint kinematics to achieve a high patient satisfaction. It can thus only be speculated if other inlay designs, such as an inlay with a flat lateral plateau or a mobile bearing inlay would lead to beneficial results for the patients, or if they are not even counterproductive. So far, when comparing fixed- and mobile-bearing systems, it has not been possible to prove in the clinical course that either of the two systems is superior [39, 40]. Furthermore, to date, there is no general evidence-based consensus to prefer the posterior stabilized or the cruciate-retaining design. Fluoroscopy findings from de Carvalho et al. (2011), who observed a higher femoral rollback in their posterior-stabilized than in their cruciate-retaining system [41]. The design of the cam-post mechanism moreover influences the femoral rollback substantially [42] possibly even leading to a medial femoral rollforward [43]. Based on intraoperative passive motion analyses, Cromie et al. suggested that such a medial femoral rollforward is generated by resecting the PCL, which is then not improved by using a posterior stabilized design [44]. In a recent meta-analysis comparing kinematic gait parameters and functional outcome, nine studies were compared finding no clinical difference between posterior stabilized and the cruciate-retaining knees with the exception of a slightly better maximum flexion in patients operated with a posterior stabilized knee [45]. Another clinical study was also not able to find significant differences between both systems [46]. In our study, the femoral rollback was almost identical for the GCR and the GPS system. Taken together, all these observations suggest that the kinematics also strongly depend of the actual implant design and configuration of the manufacturer. This would mean, that such kinematic analyses data should be made available from each manufacturer when advocating a certain way of implantation. With regard to the used system herein, in our opinion the decision should be made intraoperatively. When there is a severe posterior instability, a posterior stabilized implant is indicated. In all other cases and even in only slight posterior instabilities, we would recommend using the cruciate-retaining design.

Inherent to the design of the coupling, the SSL knee showed negligible rollback or tibial rotation. The slight remaining rotation measured in some knees is attributed to the fact that the calculated cylindrical axis was not completely congruent to the actual hinge axis of the implant in all knees. Interestingly, these results also apply for the RSL knee where at least a rotational movement would theoretically be possible. The rotational center would, however, then be in the center of the hinge and not on the medial tibia plateau. An external rotation of the femur on the tibia would thus not lead to just a lateral femoral posterior rollback but also to a medial femoral anterior rollforward. If no rotational forces are applied—as in the present study design—this mechanism does not seem to take place. This in turn implies that the forces acting on the knee joint and inducing the lateral femoral rollback in a normal knee [47] seem to be neutralized by either the design of the prosthesis or the fact that the collateral ligaments had been resected during implantation. Transferring the biomechanical results to a clinical decision-making, in our opinion the RSL knee is the implant of choice. Only in severe cases of instability, e.g. after expanded tumor resections with a lack of most soft tissue, the SSL design is needed.

Due to our experimental setup we were not able to record kinematics in the transverse plane, which is especially in hinged prostheses noteworthy. This so called telescoping is technically not possible with the implant used herein. However, the axial forces are transmitted unbuffered by the collar mechanism. Hence, in our opinion, kinematics in the transverse plane play an appreciable role in aseptic loosening. Beside joint kinematics, the sagittal position of the cylindrical femur axis on the tibia is positioned more ventrally in the two coupled prostheses than in the native knee and the uncoupled knee systems. This phenomenon is slightly more pronounced in flexion due to the lacking femoral rollback in the coupled devices. Such a ventral prominence of the femoral shield in flexion could possibly alter pressures and tensions on the patella and it may also affect the functioning of the muscles controlling the knee. Speaking in clinical terms, one can speculate that this can lead to knee pain, especially peripatellar, although the prosthesis is implanted technically correct. Since all femoral and tibial components of all different implants in this study were implanted at the identical positions on tibia and femur, we would attribute this observation to be a consequence of the relatively ventral positioning of the hinge in the tibial plateau. In the femoral component, the rotational axis of the hinge coincides well with the cylindrical axis that had been defined by the geometry of the posterior femoral condyles. It should thus also be in the correct rotational center for physiologic knee movement. In contrast, in the tibia the anchoring of the stem is more ventral, as its position also needs to follow considerations of stability of the construct and the geometry of the tibia. Since the proximal tibia is tilted dorsally in the metaphysis, this does not allow a very dorsal positioning of the pivot. Of note, this dorsiflexion of the metaphysis is imitated by the RSL tibial component with an integrated slope of 8°.

### Study limitations

To prevent tendon rupture only a reduced force level was simulated thus not reaching total physiological loads. Previous studies on the same knee simulator had, however, shown that the obtained results can be qualitatively extrapolated for higher loads [26]. Also system immanent are the use of a constant force and quasi-static conditions. Varying forces and dynamic movement can also change the kinematics of the prostheses in vivo. We also did not test rotational forces but just axial loading. For this reason, the differences of the RSL and SSL might appear smaller than they are in-vivo where often rotational forces are also present. It must also be pointed out, that the presented results only directly apply to the implant series directly tested. Although it should be possible to transfer the general concept of the results to implants from other manufacturers or knee series, numerous confounding factors might lead to slightly different results such as conformity of the inlay, geometry of the condyle (single- vs. multi-radius design), desired tibial slope, shape of the post and geometry of the coupling mechanism. One key limitation with respect to interpretation of the data is the fact that it remains still unknown to what extent a TKA should imitate the physiological joint kinematics to lead to satisfactory results.

## Conclusions

The GCR and GPS prostheses closely imitate the joint kinematics of the native knee joint. They do present, however, with a slightly reduced medial femoral rollback. The observed differences might be attributed to the inlay conformity, possibly necessary due to the lack of the anterior cruciate ligament. The coupled SSL and RSL prostheses show no femoral rollback and the RSL does not present with an external femoral rotation in flexion when no rotational additional forces are applied. For this implant series, the coupled implants present with a more ventrally located femoral axis in the tibia than their primary counterparts. The positioning of the coupling mechanism in the femoral and tibial component thus can already lead to altered joint kinematics even in prosthesis with an identical surface geometry.

## Data Availability

The datasets used and/or analyzed during the current study are available from the corresponding author on reasonable request.

## Abbreviations

ANOVA: Analysis of variance
CS: Coordinate system
CT: Computed tomography
MRI: Magnet resonance imaging
TKA: Total knee arthroplasty
